# A novel chimeric recombinant protein PDHB-P80 of *Mycoplasma agalactiae *as a potential diagnostic tool

**DOI:** 10.22099/mbrc.2020.37684.1513

**Published:** 2020-09

**Authors:** Malihe Akbarzadeh-Niaki, Abdollah Derakhshandeh, Nasrin Kazemipour, Vida Eraghi, Farhid Hemmatzadeh

**Affiliations:** 1 Department of Pathobiology, Biotechnology Section, School of Veterinary Medicine, Shiraz University, Shiraz, Iran; 2 Department of Pathobiology, School of Veterinary Medicine, Shiraz University, Shiraz, Iran; 3 Department of Basic Sciences, School of Veterinary Medicine, Shiraz University, Shiraz, Iran; 4 School of Animal and Veterinary Sciences, The University of Adelaide, SA, Australia

**Keywords:** Contagious agalactia, Serodiagnostic assay, Antigen, Recombinant fusion protein

## Abstract

The aim of this study was to construct, expression of a novel recombinant chimeric protein consisting of Pyruvate dehydrogenase beta subunit (PDHB) and high antigenic region of integral membrane lipoprotein P80 of *Mycoplasma agalactiae* as a potential diagnostic tool. The full-length sequence of *pdhb* and a portion of antigenic regions of P80 were selected and analyzed by CLC main workbench 5.5 software. Several linkers and three dimensional structure of PDHB-P80 were compared to the native PDHB and analyzed to select a proper one for expression. The fusion gene sequence was optimized and synthesized in pMAT cloning vector. The synthetic pMAT-*pdhb*-*p80* was digested using *Bam *HI and *Sal* I restriction enzymes and ligated into pMAL-p5X expression vector. The pMAL-*pdhb*-*p80 *construct was transfected into *E.coli* BL21 strain cells and expressed protein were purified using amylose resin. and the purified protein was analyzed in sodium dodecyl sulfate-polyacrylamide gel electrophoresis (SDS-PAGE) and Western blotting. *In silico* analysis demonstrated that fusion proteins using IgG4 middle hinge (CPSCP) with TM-score of 0.99 showed the higher similarity between three dimensional structure of PDHB before and after fusion with high antigenic region of P80. Successful cloning verified by PCR colony, double digestion and sequence analysis. Besides, SDS-PAGE analysis and Western blotting indicated and confirmed the expression of intact recombinant chimeric protein MBP-PDHB-P80 along with some truncated forms of the recombinant protein. it could be concluded that the fusion construct has a potential for serodiagnostic assay in future studies.

## INTRODUCTION

*Mycoplasma agalactiae* is a cell wall-less bacteria identified as the classical agent of contagious agalactia (CA) which is a worldwide serious syndrome affecting sheep and goats and responsible for severe economic losses to dairy industry [1-3]. Regarding the potential risk of disease durability and chronicity due to the transmission of the pathogen via asymptomatic carriers, the issue of diagnosis has grown in importance [4, 5]. Recent developments in the field of recombinant proteins have led the researchers to an interest in analysis the antigenic proteins such as P30, P40, P48, P55 and P80 of *Mycoplasma agalactiae* in order to find out a diagnostically relevant marker [6-10]. The studies carried out on P80 demonstrated the presence of this integral membrane lipoprotein at the first symptoms of infection and its conservation among *Mycoplasma agalactiae* strains [9, 11] which makes it a proper candidate for developing an efficient serodiagnostic assay. Pyruvate dehydrogenase beta subunit (PDHB) is considered as a dual functional phosphoprotein, playing a vital role in metabolic pathway along with the pathogen attachment to the host cell surface. Until now, PDHB is identified as an immunoreactive protein in some *Mycoplasma* species such as *Mycoplasma capricolum subsp. capripneumoniae*, *Mycoplasma hyopneumoniae* and *Mycoplasma bovis* [12-14]. The purpose of this study was to express a novel chimeric recombinant protein consisting of PDHB and high antigenic region of P80. Since the three dimension structure of the antigen is an important issue for its immune-reactivity, we evaluated three dimension structure of PDHB in designed chimeric protein compared with native PDHB and then expressed the desired fusion protein in *E. coli* as host.

## MATERIALS AND METHODS

**Antigens selection: **Sequences registered at *National Center for Biotechnology Information* (NCBI) for *pdhb* gene (GenBank no. CBH40332.1, 984 bp) and C-terminal of *p80* gene (GenBank no. AF299294 ,1011 bp) were analyzed. Assessment of high antigenic part of P80 among the full length sequence of gene was done using CLC main workbench 5.5 software.

**Construction of fusion gene** ***pdhb-p80*** **with conserving of three dimensional structure of PDHB protein: **For better folding of PDHB, 39 natural linkers and 18 artificial linkers (listed in supplementary information) were tried to be introduced between two genes [15]. Also, secondary structure of PDHB for determination of the best composition of linker amino acids, was evaluated by assessment of each protein before and after fusion with linker using CLC software. Three dimensional structure of PDHB and chimeric fusion protein (PDHB-P80) to obtain PDB (protein data bank) files, was done using Protein Homology/analogY Recognition Engine (Phyre2) http://www.sbg.bio.ic.ac.uk/~phyre2/html/page.cgi?id=index). Then alignment of three dimensional structure of PDHB-P80 with native PDHB was analyzed using TM-align based on achieved TM-scores (http://zhanglab.ccmb.med.umich.edu/TM-align) [16].

**Plasmid designing: **The designed fusion gene, conserving the three dimensional structure of PDHB, has been constructed. Since the universal stop codon TGA is translated to tryptophan in Mycoplasma [17], the sequence was scanned for this genetic code and one TGA converted to alternative stop codon TGG. Besides, six-HIS tag-encoding was inserted at N-terminal and the restriction sites of *Bam *HI and *Sal* I were added at 5′ and 3′ ends, respectively. Nucleotide sequence of fusion gene was optimized for the proper expression in *E. coli* and synthesized in pMAT cloning vector by Thermo Fisher Scientific Company (USA).

**Cloning procedure:** *E. coli* BL21 was grown at 37ºC in 2X Yeast Tryptone (2YT) glucose broth (16 g Tryptone, 5 g, NaCl, 10 g yeast extract and 0.1% D-Glucose per liter) supplemented with ampicillin (100 mg/ml). The synthetic pMAT-PDHB-P80 was electroporated into *E.coli* BL21 strain using Bio-Rad Gene Pulsar Xcell™ electroporation system (Bio-Rad Laboratories, Inc USA). 100μg/ml ampicillin was used to select the recombinant transformants. After amplification, the plasmid was extracted, then digested with restriction enzymes *Bam *HI and *Sal* I (Invitrogen Anza^TM^, Thermo Fisher Scientific, USA). Same enzymes were used for digestion of pMAL-p5X expression vector producing compatible sticky ends. Digested vector and insert resolved in a 0.75% agarose gel after electrophoresis and purified using QIAquick Gel Extraction kit (QIAGEN, Germany), then ligated by Anza^TM^T4 DNA ligase master mix (Thermo Fisher Scientific, USA) according to the manufacturer's instructions. Finally, the constructed recombinant expression vector of PDHB-P80-pMAL were transformed into fresh electrocompetent *E.coli* BL21. At the next step, the obtained colonies were verified to contain PDHB-P80-pMAL using double digestion and PCR colony using pMAL-p5X expression vector primers (F:5´-GGT CGT CAG ACT GTC GAT GAA GCC-3´and R:5´ TGT CCT ACT CAG GAG AGC GTT CAC-3´) with an annealing temperature of 55ºC. Also, Sanger sequencing analysis (Australian Genome Research Facility, AGRF) confirmed the positive recombinant colonies [18].

**Protein expression and western blotting:** Overnight cultures of positive recombinant bacteria were diluted 1:10 in a total volume of 100ml of 2YT containing ampicillin and incubated at 37ºC with shaking at 180 rpm to optical density (OD)_600_ of 0.7. Protein expression was induced with 0.5 mM isopropyl-b-D-thiogalactopyranoside (IPTG) and after 4 hours incubation at 25ºC under agitation, the culture fluid was centrifuged at 4000g for 15 minutes and the pelleted cells were obtained. The whole cell proteins were analyzed by sodium dodecyl sulfate-polyacrylamide gel electrophoresis (SDS-PAGE) with 10% polyacrylamide gels followed by Coomassie Blue staining. A non-induced culture was included as negative control. The apparent molecular weight of the expressed protein were determined using SMOBIO PM1500 (*Taiwan*) protein marker. To conform the expression of the recombinant chimeric protein, the proteins were run in SDS-PAGE then transferred on to a nitrocellulose membrane using a Trans-Blot semidry apparatus (Bio-Rad, U.S.A) as described by the manufacturer. The membrane was blocked by 10% bovine serum albumen (BSA) in PBS containing 0.05% Tween 20 for overnight under rotating. After several washing with PBST, Blots were incubated for 1 hour at 37°C with 1:1000 Anti-poly Histidine-HRP antibody (Sigma, A7058). After three more washes, protein bands were visualized using diaminobenzidine (DAB) (Sigma-Aldrich Pty. Ltd) as substrate [18, 19].

## RESULTS

The full length of *pdhb* and 480 bp as high antigenic region of the *p80* gene were selected. 

**Figure 1:**
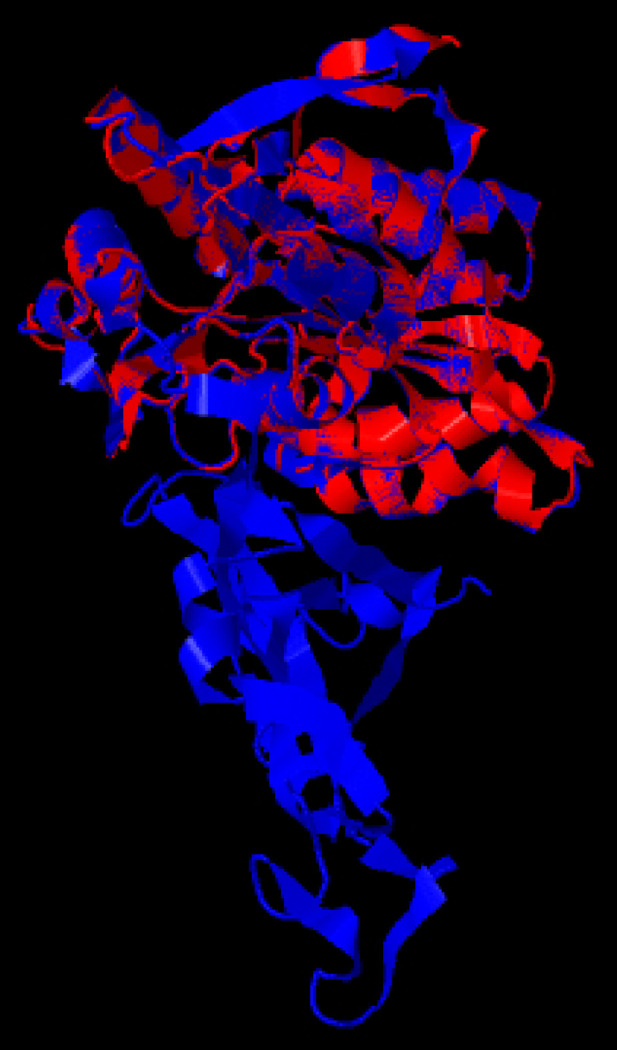
Three dimension structure alignment of chimeric protein with native form of PDHB. Alignment of PDHB-P80 (in blue) with native PDHB (in red).

After assessment of all results for analyzing alignment of three dimension structure of PDHB protein before and after fusion with high antigenic region of P80 depended on different linkers between them, IgG4 middle hinge (CPSCP) was subsequently used as linker with TM-score of 0.99 (Fig. 1).

The synthetic fusion gene *pdhb-p80* was successfully cloned into the expression vector pMAL-p5X and constructed *mbp-pdhb-p80* fusion gene. Then, the obtained colonies were verified to contain *pdhb-p80-*pMAL by PCR colony and double digestion and confirmed by sequencing in both directions.

**Figure 2:**
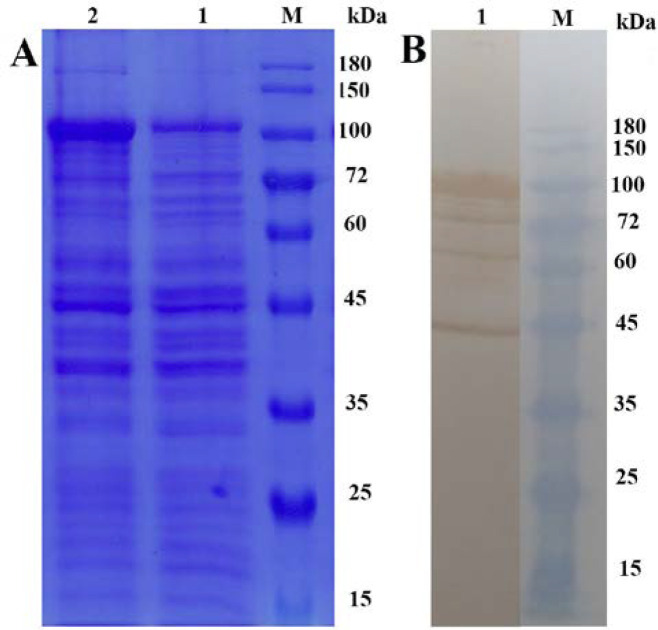
**A:** SDS-PAGE analysis showing the expression of 96 kDa recombinant protein compared negative control, Lane M: SMOBIO PM1500 protein marker, Lane 1: non-induced cells, Lane 2: 0.5 mM IPTG-induced cells after 4 hours at 25ºC. **B:** Western blotting analysis using Anti-poly Histidine-HRP antibody confirming the expression of 96 kDa recombinant protein along with the truncated forms of recombinant protein around 45, 62 and 72 kDa

According to the predicted MBP-PDHB-P80 protein molecular mass, approximately 96.5 kDa, a proper expression was observed after IPTG induction on Coomassie blue stained SDS-polyacrylamide gel, which was not existed in uninduced cells (Fig. 2A). 30μl of whole induced cells were used for western blotting and the Anti-poly Histidine-HRP antibody specifically reacted with the histidine tag of the expressed recombinant chimeric protein. The protein band around 96.5 kDa is corresponded to the expected recombinant protein containing 54 kDa PHDB-P80 and 42.5 kDa MBP. However, three more bands about 45, 62 and 72 kDa have been observed that are probably the truncated forms of MBP-PDHB-P80 protein. The presence of these bands confirmed the expression of a less amount of truncated MBP-PDHB-P80 protein along with the intact one (Fig. 2B).

## DISCUSSION

Contagious agalactia caused by *Mycoplasma agalactiae* is a worldwide multifactorial syndrome in goat and sheep [3]. Several researches demonstrated the presence of this agent in both naturally and experimentally infected carriers several months after the acute stage of infection, leads to disease persistence and chronicity, which could be an important threat for dairy industry [4, 5, 20]. Hence, considerable attention has been paid to evaluation of antigenic proteins of *Mycoplasma agalactiae* for diagnostic applications to control this disease [6-10]. In this study, we designed, cloned and expressed a chimeric recombinant protein consisting of PDHB and high antigenic region of P80 for further immunogenicity evaluation. To our knowledge, this is the first study to propose PDHB in *Mycoplasma agalactiae*, although, cell surface location and the antigenicity of this protein has been confirmed in some other mycoplasma species such as, *Mycoplasma capricolum subsp. Capripneumoniae, Mycoplasma hyopneumoniae* and *Mycoplasma bovis* [12-14]. We selected P80 to construct the fusion protein based on the findings obtained in Tola (2001) and Fusco (2007) investigations, which proved the antigenicity, stability and conservation of this lipoprotein in *Mycoplasma agalactiae* and suggested it as a potential serodiagnostic marker [8, 9]. Two protein were fused via IgG4 middle hinge (CPSCP) with TM-score of 0.99 demonstrating a high similarity index between three dimension structure of PDHB before and after fusion with high antigenic region of P80. These findings suggest a great potential of this chimeric protein for serodiagnostic applications regarding the conservation of three dimension structure of PDHB. Sun *et al*. established an indirect ELISA using recombinant PDHB as coating antigen and suggested it as a suitable approach for antibody detection in *Mycoplasma bovis* [14] Their finding is in agreement with our estimation.

The molecular weight of expressed protein indicated in western blotting analysis is 96.5 kDa, which is consistent with the estimated molecular weight by CLC software analysis; however, there have been other 45, 62 and 72 kDa protein bands. It is common especially for large proteins that we have multiple bands due to protein truncation. Truncated proteins are due to protein degradation or premature termination in ribosomes [21]. We can conclude that breaking down of our large 96.5 kDa recombinant protein results in these truncated forms of protein, which retain the histidine tag specifically reacting with Anti-poly Histidine-HRP antibody. Several studies on expression of recombinant MBP fusion proteins faced this issue and considered it as a normal situation [18, 22].

Western blot analysis confirmed the expression of our constructed recombinant protein PDHB-P80 using Anti-poly Histidine-HRP antibody, whereas, clearly further research including the protein purification and testing the reaction of infected sera with recombinant PDHB-P80 will be required to validate the immunologic reactions of the recombinant protein. Summing up, we proposed a novel designed chimeric protein of *Mycoplasma agalactiae* as a potential marker for serodiagnostic assays. Moreover, the next stage of our research will be protein purification and experimental confirmation of immunogenicity of our novel fusion protein.

## Conflict of Interest:

The authors declare that they have no conflict of interest. 

## Supplementary Materials

Supplement
