# Supplementary material for: A novel chimeric recombinant protein PDHB-P80 of Mycoplasma agalactiae as a potential diagnostic tool
Source: Mol Biol Res Commun. 2020 Sep;9(3):123–8. doi: 10.22099/mbrc.2020.37684.1513 (PMC7727766; doi:10.22099/mbrc.2020.37684.1513)
Supplement: Supplement [file mbrc-9-123-s001.pdf]

Supplementary information

Natural linker from patent search

SynLinker Entry		PDB sequence		Linker Information						Structure	Selected Linkers
Accession	Name	Start	End	ID	Residues	Angstrom	Solvent Accessibility	Flexibility	Sequence	Show/Hide	Add/Remove
NP_1	(US4946778A) A helical segment from human hemoglobin Linker 1	-	-	1	22	24.866	-	11.657	AQGTLSPADKTNVKAAWGKVMT	show	
NP_10	(US6165476A) Human IgG3 Middle Hinge Linker 2	-	-	1	42	46.633	-	24.576	EPKSCDTPPCPRCPEPKSCDTPPCPRCPEPKSCDTPPCPRCP	show	
NP_11	(US6165476A) Human IgG3M15 Middle Hinge Linker 1	-	-	1	11	17.115	-	5.5152	CDTPPPCPRCP	show	
NP_12	(US6165476A) Human IgG4 Upper Hinge Linker 1	-	-	1	7	5.544	-	4.9091	ESKYGPP	show	
NP_13	(US6165476A) Human IgG4 Middle Hinge Linker 1	-	-	1	5	12.172	-	2.202	CPSCP	show	
NP_14	(US6165476A) Human IgG4 Lower Hinge Linker 1	-	-	1	8	17.439	-	4.3232	APEFLGGP	show	
NP_15	(US6165476A) Mouse IgG1 Upper Hinge Linker 1	-	-	1	6	15.258	-	2.8737	VPRDCG	show	
NP_16	(US6165476A) Mouse IgG1 Middle Hinge Linker 1	-	-	1	7	17.794	-	2.3485	CKPCICT	show	
NP_17	(US6165476A) Mouse IgG1 Lower Hinge Linker 1	-	-	1	6	10.07	-	3.399	VPSEVS	show	
NP_18	(US6165476A) Mouse IgG2A Upper Hinge Linker 1	-	-	1	9	20.641	-	5.9091	EPRGPTIKP	show	
NP_19	(US6165476A) Mouse IgG2A Middle Hinge Linker 1	-	-	1	7	12.455	-	3.2273	CPPCKCP	show	
NP_2	(US6165476A) Human IgG1 Upper Hinge Linker 1	-	-	1	10	21.628	-	6.4192	EPKSCDKTHT	show	
NP_20	(US6165476A) Mouse IgG2A Lower Hinge Linker 1	-	-	1	8	20.619	-	4.1919	APNLLGGP	show	
NP_21	(US20100221778A1) Cellulase Linker 1	-	-	1	36	46.054	-	21.126	PGNPTTTVVPPASTSTSRPTSSTSSPVSTPTGQPGG	show	
NP_22	(US20100221778A2) Cellulase Linker 2	-	-	1	35	12.618	-	22.409	PDGGSGNPNPPVSSSTPVPSSSTTSSGSSGPTGGT	show	

NP_23	(US20100221778A3) Cellulase Linker 3	-	-	1	24	41.493	-	14.985	GTTPNPPASSSTTGSSTPTNPPAG	show	☐
NP_24	(US20100221778A4) Cellulase Linker 4	-	-	1	33	48.362	-	19.722	PGAGNGGNNGGNPPPPTTTTSSAPATTTTASAG	show	☐
NP_25	(US20100221778A5) Cellulase Linker 5	-	-	1	26	26.232	-	15.576	GGNPPGGNRGTTTTRRPATTTGSSPG	show	☐
NP_26	(US20100221778A6) Cellulase Linker 6	-	-	1	25	43.048	-	13.677	TGTGTGTGTGTGTGTGTTTSSAPAA	show	☐
NP_27	(US20100221778A7) Cellulase Linker 7	-	-	1	34	44.24	-	22.374	GSSGTPPSNPSSSASPTSSTAKPSSTSTASNPSG	show	☐
NP_28	(US20100221778A8) Cellulase Linker 8	-	-	1	33	42.871	-	19.157	GTSTGGSTTTTASGTTSTSKASTTSTSSTSTGTG	show	☐
NP_29	(US20100221778A9) Cellulase Linker 9	-	-	1	33	29.358	-	19.394	TVSSSSVSSSHSSTSTSSSHSSSSTPPTQPTGV	show	☐
NP_3	(US6165476A) Human IgG1 Middle Hinge Linker 1	-	-	1	5	9.1	-	2.2172	CPPCP	show	☐
NP_30	(US20100221778A10) Cellulase Linker 10	-	-	1	48	33.725	-	28.298	PSSGGTSSSSSAAPQSTSTKASTTTSAVRTTSTATTKTTSSAPAQGTN	show	☐
NP_31	(US20100221778A11) Cellulase Linker 11	-	-	1	27	30.195	-	17.232	GGNPPGGNPPGTTTPRPATSTGSSPGP	show	☐
NP_32	(US20100221778A12) Cellulase Linker 12	-	-	1	41	52.339	-	23.354	ASSSSSTRAASTTSRVSPTTSRSSSATPPPGSTTTRVPPVG	show	☐
NP_33	(US20100221778A13) Cellulase Linker 13	-	-	1	25	21.021	-	15.177	PPPPPASSTTFSTTRRSSTTSSSPS	show	☐
NP_34	(US 20120034160A1) 5-13 amino acids from the N termini of human Ck and CH1 domains Linker 1	-	-	1	6	14.995	-	3.9444	QPKAAP	show	☐
NP_35	(US 20120034160A2) 5-13 amino acids from the N termini of human Ck and CH1 domains Linker 2	-	-	1	5	12.76	-	2.1465	TVAAP	show	☐
NP_36	(US 20120034160A3) 5-13 amino acids from the N termini of human Ck and CH1 domains Linker 3	-	-	1	6	17.231	-	3.9646	ASTKGP	show	☐
NP_37	(US 20120034160A4) 5-13 amino acids from the N termini of	-	-	1	13	36.388	-	6.9444	QPKAAPSVTLFPP	show	☐

	human Ck and CH1 domains Linker 4										
NP_38	(US 20120034160A 5) 5-13 amino acids from the N termini of human Ck and CH1 domains Linker 5	-	-	1	12	32.844	-	4.6919	TVAAPSVFIFPP	show	
NP_39	(US 20120034160A 6) 5-13 amino acids from the N termini of human Ck and CH1 domains Linker 6	-	-	1	13	38.002	-	6.899	ASTKGPSVFPLAP	show	
NP_4	(US6165476A) Human IgG1 Lower Hinge Linker 1	-	-	1	8	13.381	-	4.4242	APELLGGP	show	
NP_5	(US6165476A) Human IgG2 Upper Hinge Linker 1	-	-	1	3	7.083	-	2.4848	ERK	show	
NP_6	(US6165476A) Human IgG2 Middle Hinge Linker 1	-	-	1	9	17.959	-	3.3333	CCVECPPCP	show	
NP_7	(US6165476A) Human IgG2 Lower Hinge Linker 1	-	-	1	7	17.869	-	3.7828	APPVAGP	show	
NP_8	(US6165476A) Human IgG3 Upper Hinge Linker 1	-	-	1	12	25.683	-	6.5859	ELKTPLGDTTHT	show	
NP_9	(US6165476A) Human IgG3 Middle Hinge Linker 1	-	-	1	5	9.56	-	2.0101	CPRCP	show	

Artificial linker from patent search

SynLinker Entry		PDB sequence		Linker Information						Structure	Selected Linkers
Accession	Name	Start	End	ID	Residues	Angstrom	Solvent Accessibility	Flexibility	Sequence	Show / Hide	Add / Remove
AP_1	(US4946778 A) Double Linker 1	-	-	1	3	7.018	-	2.0909	PGS	show	☐
AP_10	(US5525491 A) Serine-rich Linker 6	-	-	1	13	11.15	-	9.096	SGSSSSGSSSSGS	show	☐
AP_11	(US5525491 A) Serine-rich Linker 7	-	-	1	19	26.2	-	11.99	SVTVSSSGSSSSGSSSSGS	show	☐
AP_12	(US5856456 A) Linker 1	-	-	1	18	40.278	-	12.707	GSTSGSGKPGSGEGSTKG	show	☐
AP_13	(US5856456 A) Linker 2	-	-	1	18	16.437	-	12.232	GSTSGSGRPGSGEGSTKG	show	☐
AP_14	(WO2012083424A1) Linker 1	-	-	1	3	6.27	-	1.8737	ATK	show	☐
AP_15	(WO2012083424A2) Linker 2	-	-	1	3	6.713	-	2.1212	ASK	show	☐
AP_16	(WO2012083424A3) Linker 3	-	-	1	6	11.199	-	3.9949	ATKASK	show	☐
AP_17	(WO2012083424A4) Linker 4	-	-	1	7	9.835	-	4.3889	ATKGATK	show	☐
AP_18	(US20130280285A1) Modified hinge region of the human CD8 alpha-chain Linker 1	-	-	1	62	33.315	-	30.182	ALSNSIMYFSHFVPVFLPAKPTTTPAPRPPTPAPT IASQPLSLRPEASRPAAGGAVHTRGLD	show	☐
AP_2	(US4946778 A) Double Linker 2	-	-	1	7	9.931	-	3.7071	IAKAFKN	show	☐
AP_3	(US4946778 A) Single Linker 1	-	-	1	18	31.742	-	10.753	KESGSVSSEQLAQFRSLD	show	☐
AP_4	(US4946778 A) Single Linker 2	-	-	1	14	28.412	-	5.101	VRGSPAINVAVHVF	show	☐
AP_5	(US5525491 A) Serine-rich Linker 1	-	-	1	5	9.382	-	3.5101	SSSSG	show	☐
AP_6	(US5525491 A) Serine-rich Linker 2	-	-	1	10	16.173	-	7.0202	SSSSGSSSSG	show	☐
AP_7	(US5525491 A) Serine-rich Linker 3	-	-	1	15	18.03	-	10.53	SSSSGSSSSGSSSSG	show	☐
AP_8	(US5525491 A) Serine-rich Linker 4	-	-	1	20	33.484	-	14.04	SSSSGSSSSGSSSSGSSSSG	show	☐
AP_9	(US5525491 A) Serine-rich Linker 5	-	-	1	25	40.363	-	17.551	SSSSGSSSSGSSSSGSSSSGSSSSG	show	☐

TM-align Results

```
 **************************************************************************
 *                        TM-align (Version 20170708)                     *
 * An algorithm for protein structure alignment and comparison            *
 * Based on statistics:                                                   *
 *       0.0 < TM-score < 0.30, random structural similarity              *
 *       0.5 < TM-score < 1.00, in about the same fold                    *
 * Reference: Y Zhang and J Skolnick, Nucl Acids Res 33, 2302-9 (2005)    *
 * Please email your comments and suggestions to: zhng@umich.edu          *
 **************************************************************************

Name of Chain_1: A935253
Name of Chain_2: B935253
Length of Chain_1:  328 residues
Length of Chain_2:  493 residues

Aligned length=  328, RMSD=   0.48, Seq_ID=n_identical/n_aligned= 1.000
TM-score= 0.99481 (if normalized by length of Chain_1)
TM-score= 0.66286 (if normalized by length of Chain_2)
(You should use TM-score normalized by length of the reference protein)
```
